# Pilot evaluation of a novel unilateral onychectomy model and efficacy of an extended release buprenorphine product

**DOI:** 10.1186/s12917-017-0943-5

**Published:** 2017-01-24

**Authors:** Masataka Enomoto, Patricia D. Kigin, David Bledsoe, Robyn Slone, Jonathan Hash, Charles E. Smith, B. Duncan X. Lascelles

**Affiliations:** 1 Comparative Pain Research Laboratory Department of Clinical Sciences, College of Veterinary Medicine, North Carolina State University, Raleigh, NC USA; 2 Farnam Companies, Inc., Phoenix, AZ USA; 3 Present Address: Qualitas BioSciences, LLC, Peoria, AZ USA; 4 Department of Statistics, North Carolina State University, Raleigh, NC 27695 USA; 5 Comparative Medicine Institute, North Carolina State University, Raleigh, NC 27606 USA; 6 Center for Pain Research and Innovation, UNC School of Dentistry, Chapel Hill, NC USA

**Keywords:** Buprenorphine, Extended release, Pressure sensitive walkway, Landing, Cat, Kinetic

## Abstract

**Background:**

Non-steroidal anti-inflammatory drugs (NSAIDs), transdermal fentanyl patches, and transmucosal buprenorphine are probably the most commonly used options for providing post-operative analgesia in the early at-home period. However, these require daily administration or are associated with abuse concerns. One of the significant unmet needs in veterinary surgery and pain management is for longer acting opioids for cats to effectively bridge the gap between the in-hospital and at-home recovery periods.

A proof of concept study of an extended release formulation of buprenorphine HCL (ER-Bup) was conducted using objective kinetic measures and a unilateral onychectomy model. Using a blinded, randomized, two period crossover design, four cats were allocated to control (saline) or ER-Bup (0.6 mg/kg, subcutaneously [SC]) treatment groups. All animals underwent a unilateral forelimb onychectomy per period with a washout/recovery period in between. Observational pain scores and kinetic data (using a pressure sensitive walkway [PSW]) were collected prior to (baseline) and at intervals for 72 h following surgery. Symmetry indices were derived for kinetic variables (peak vertical force [PVF]; vertical impulse [VI]) of each forelimb for landing following a jump and for walking. A rescue analgesic protocol was in place. Effect of surgery and treatment were evaluated using a mixed model statistical approach.

**Results:**

No cats required rescue analgesics based on subjective pain score. ER-Bup had a positive influence on subjective pain scores during the 72 h postsurgery (*p* = 0.0473). PVF and VI of the operated limb were significantly decreased for both landing (*p* < 0.0001 and *p* < 0.0001) and walking (*p* < 0.0001 and *p* < 0.0001 respectively) compared to control. ER-Bup resulted in significantly decreased asymmetry in limb use during landing (PVF, *p* < 0.0001; VI, *p* < 0.0001) and walking (PVF, *p* = 0.0002, VI, *p* < 0.0001). The novel use of data collected following a jump from an elevated platform appeared to provide all desired information and was easier to collect than walking data.

**Conclusion:**

This study demonstrates that SC administration of ER-Bup may be an effective analgesic for a 72 h period postoperatively. Furthermore, landing onto a PSW from an elevated perch may be a useful and efficient way to assess analgesics in cats using a unilateral model of limb pain.

**Electronic supplementary material:**

The online version of this article (doi:10.1186/s12917-017-0943-5) contains supplementary material, which is available to authorized users.

## Background

Inadequate control of pain in the perioperative period in cats has been linked to poor recoveries, postoperative complications and potentially chronic pain [1]. Non-steroidal anti-inflammatory drugs (NSAIDs), transdermal fentanyl patches, and transmucosal buprenorphine are probably the most commonly used options for providing post-operative analgesia in the early at-home period [2]. However, there are concerns about side effects of NSAIDs; there are potential abuse concerns with sending fentanyl patches into the home environment [3]; and transmucosal buprenorphine is not always easy for owners to accomplish [2]. One of the significant unmet needs in veterinary surgery and pain management is for longer acting opioids for cats to effectively bridge the gap between the in-hospital and at-home recovery periods.

Buprenorphine, a synthetic opioid drug that is classified as a partial μ opioid receptor agonist, is commonly used for pain management in cats via multiple administration routes [4, 5]. This drug reportedly has a strong affinity for, and dissociates slowly from, the mu opioid receptors [6]. Because of this pharmacodynamic profile, the immediate release formulation of buprenorphine is considered to be one of the longest-acting opioids available with a duration of action greater than 6 h [7, 8]. Additionally, the incidence of undesirable side effects, such as vomiting, nausea, respiratory depression and dysphoria, is reportedly lower compared to other opioids [2]. Due to these characteristics, it has become one of the most popular opioid analgesics for use in cats in many countries [9, 10]. A recent clinical study, however, did illustrate that cats undergoing ovariohysterectomy may require a second dose of buprenorphine 4 h after surgery [11]. This, and other data, suggests that a single-injection is not sufficient for effective postoperative pain relief [5, 12].

It is well established that it is difficult to recognize and measure pain in cats [1, 13]. Multiple methods attempting to accurately assess pain have been described [14, 15], and several research groups are in the process of developing and validating subjective pain scales for clinical use [14, 16–18]. One objective method to evaluate pain is gait analysis, which is only relevant if the origin of the pain affects gait. Pressure sensing platforms, or pressure sensitive walkways (PSW) have been investigated for the measurement of limb use in cats [19, 20] and used for the evaluation of limb use following onychectomy [21]. It is difficult to collect high quality limb use data when relying solely on cats freely walking across a PSW in a straight line (unpublished observations).

Several years ago kinetic evaluation of jumping cats using a PSW was proposed by our pain research group at NC State [20] and recently revisited by another team of researchers [19]. One potential use of this method is to measure the left-right difference in kinetic parameters when there is unilateral forelimb pain.

The availability of an extended release formulation of buprenorphine (ER-Bup) may provide effective analgesia in the immediate postoperative period in the hospital and at-home. Evaluation of an extended release formulation of buprenorphine in dogs undergoing arthrotomy provided pain relief over a 72 h period [22]. In cats, it has been reported that the administration of a single dose of ER-Bup had a similar efficacy (as evaluated by subjective pain assessment scales) and adverse effect profile as twice-daily oral transmucosal administration of buprenorphine [23]. However, there was no placebo control group in this study, and the investigators did not use a validated subjective assessment tool, and so the efficacy of the new formulation of buprenorphine is unknown.

The aim of the present study was to perform a pilot proof of principle pilot study of ER-Bup using objective kinetic measures collected from walking, and landing following a jump from an elevated perch (0.7 m), in a unilateral onychectomy model. It was hypothesized that unilateral onychectomy would result in asymmetry of forelimb kinetic values in cats, and that ER-Bup would decrease this asymmetry. Further, it was hypothesized that collecting data from cats landing from an elevated platform (jumping down) would result in measureable objective parameters similar to the classical collection of kinetic variables in freely ambulating cats.

## Methods

### Study design

The study was an experimental, masked, randomized, placebo controlled, two period cross-over design. The investigator, surgeon and any others involved in making assessments regarding product efficacy or safety were unaware of treatment assignment. The study monitor and dose administrator were un-masked but were not involved in clinical assessments. The cats were housed in a climate-controlled facility in compliance with the U.S Animal Welfare Act. All study procedures were approved by the Institutional Animal Care and Use Committee (protocol number PLRS 1006). The study was initiated at Professional Laboratory and Research Services, Inc.

#### Animals

Four purpose-bred mature domestic short-hair sexually intact two male and two female cats were used. They were considered healthy based on physical examination, complete blood count, serum biochemical analysis, and urinalysis. Body weights ranged from 2.2 to 3.2 kg. Age of all cats was approximately 10 months old. The cats were housed individually in a room with a controlled environment and were acclimated to their surroundings for at least 30 days.

### Treatment and surgical procedures

**Fig. 1:**
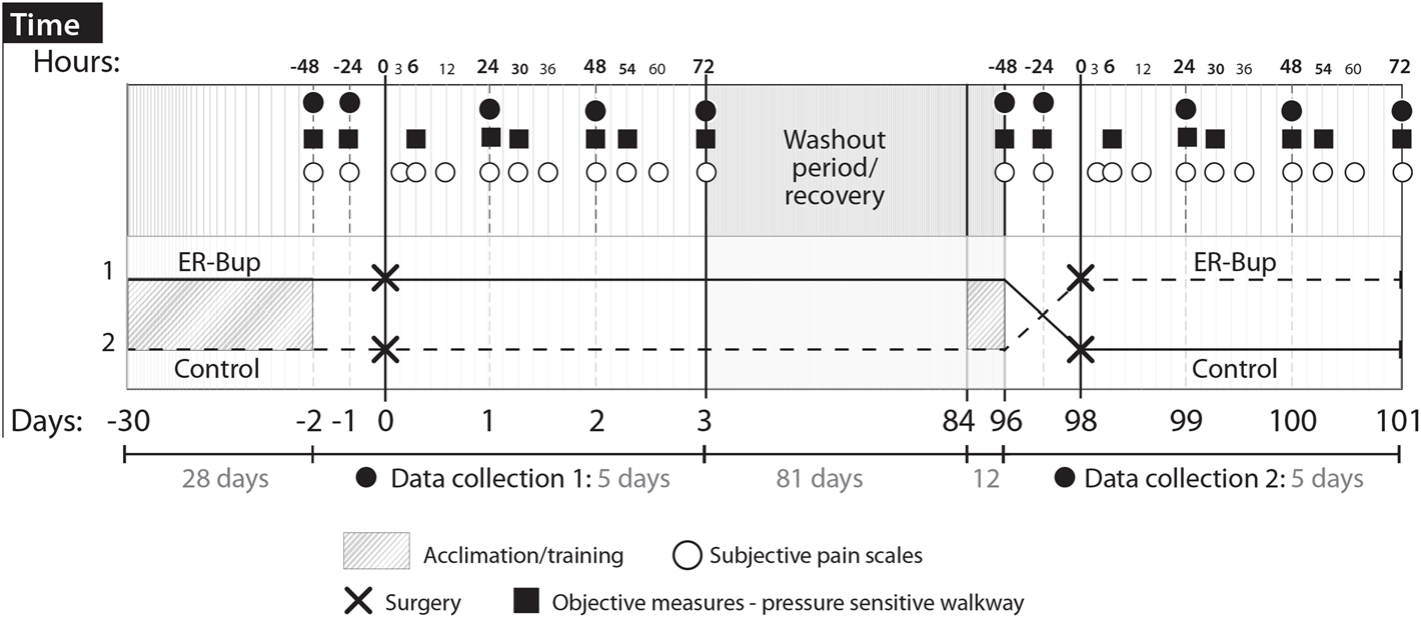
Schematic outline of the study protocol. Using a blinded, randomized, two period crossover design, four cats were allocated to control (saline) or ER-Bup (0.6 mg/kg, subcutaneously) treatment groups, in random order. The cats underwent a unilateral forelimb onychectomy per period with a washout/recovery period between. Observational pain scores and kinetic data were collected prior to (baseline) and at intervals for 72 h following surgery (total of 5 days of data collection). All cats underwent daily walking and jumping training on the PSW for approximately 30 days prior to study initiation. Following the washout/recovery period training sessions were again performed for 12 days prior to surgery. The washout/recovery period was 81 days

Treatment groups consisted of a control group (0 mg/kg; saline) and ER-Bup group (0.6 mg/kg subcutaneously, SC). In previous work (unpublished kinetic studies) 0.6 mg/kg of ER-Bup was confirmed not to cause significant ataxia, weakness or other impairment that might be interfere with ability to assess gait or other treatment during the development of this product. The cats were randomly allocated to one of the two treatments for each limb. A two period crossover study was employed to evaluate the two groups as shown in Table 1. All animals underwent two onychectomy surgeries; the first foot during period 1 and the other foot during period 2 with a washout/recovery period between (Fig. 1). Cats were deemed ready for a second surgery when peak vertical force measurements (PVF), both at a walk and following landing from an elevated platform, were within the 95% confidence interval of the average of the two baseline PVF measurements. The same anesthetic regimen and surgical procedure was used in each period. As previously described [24], guillotine-type nail clippers were carefully positioned to completely remove the third phalanx. Treatments (saline or ER-Bup) were administered 20–60 min prior to anesthetic induction. Four weeks after the first surgery, during the washout/recovery period, kinetic variables were collected at pre-determined intervals and analyzed. Upon the conclusion of the study, each cat had had both forelimbs declawed with one foot being done with the test article being incorporated into the pre-operative anesthetic procedure and the other being done with a negative control.

**Table 1:** Animal allocation and randomization schedule

Cat	Treatment sequence	Period.1		Period.2	
		Test article	Foot	Test article	Foot
1	1	Saline	L	ER-Bup	R
2	1	Saline	R	ER-Bup	L
3	2	ER-Bup	R	Saline	L
4	2	ER-Bup	L	Saline	R

*ER-Bup* extended release formulation of buprenorphine HCL

#### Anesthesia protocol

Pre-anesthetic medication with acepromazine at 0.03 mg/kg intramuscularly 20–60 min prior to anesthetic induction (administered at the same time as the ER-Bup or saline). Intravenous propofol (2–6 mg/kg) was administered to effect to induce general anesthesia. Isoflurane carried in O_2_ was administered via an endotracheal tube for the maintenance of anesthesia. During anesthesia, cats were monitored for heart rate, respiration rate, temperature, end tidal CO_2_, oxygen saturation, and blood pressure was measured noninvasively. Vital signs and comfort were periodically assessed after anesthesia until the cats were fully recovered from anesthesia and their rectal temperature was normal.

#### Collection of kinetic variables using a Pressure Sensitive Walkway (PSW)

A PSW (7100 QL Virtual Sensor 4Mat System, Tekscan, Boston, MA), 2.4 m × 0.5 m with 4 sensels/cm^2^, was used to collect kinetic data from walking and landing cats as described previously [20].

The data were recorded at 6, 24, 30, 48, 54, and 72 h after surgery. Baseline gait analysis was performed for each animal 24 and 48 h prior to each surgical procedure (Fig. 1). A 50 psi sensor range was selected following a review of previous work that indicated this was the most appropriate sensor range [20]. The output data recorded was analyzed using proprietary Tekscan software (Walkway v7.0 software, Tekscan, Boston, MA). Each day, the whole area of the pressure platform was loaded and unloaded by walking on it (conditioning), and it was then equilibrated and calibrated according to the manufacturer’s instructions. For all data collection, the frame acquisition rate was set to the maximum frequency of 60 Hz. All cats underwent daily walking and jumping down training on the PSW for approximately 30 days prior to study initiation. Following the washout/recovery period training sessions were again performed for 12 days prior to the second surgery. For the collection of walking data, valid data movies were defined as the cat traversing the PSW in a straight line, within individualized velocity and acceleration parameters, with no visually detectable movement of the head from side to side, and no visually detectable slowing down or acceleration. Cats were encouraged to traverse the mat spontaneously using treats and toys. Cardboard barriers were sometimes used on either side of the PSW to prevent the cats from wandering off the mat. Ten sets of walking data were collected, with at a target velocity of 0.6 ± 0.2 m/s and within acceleration changes of ±0.1 m/s^2^. Either using manual analysis or automated analysis available within the software, the velocity of each ‘run’ was calculated to ensure there were ten data sets collected within the velocity parameters set. As described later, post-collection analysis used the five data sets that were closest to the target velocity and acceleration parameters to minimize the impact of differences in velocity on kinetic parameters. Additionally, at each time point, ten sets of data were collected with the cats landing on the mat after jumping down from an elevated platform. The cats were encouraged to jump down onto the PSW, using treats and toys, from an elevated platform that was 0.7 m vertical distance from the ground (and PSW), landing on the PSW with their forelimbs first (landing data). Valid data movies were defined as those where cats jumped down in a forward direction, and landed with the forelimb(s) first. Using the software, kinetic data were collected from each forelimb footfall for each of five valid trials. PVF and vertical impulse (VI) were collected. All forces were normalized to and expressed as a percentage of the cat’s body weight. Forelimb symmetry indices (SI) for PVF and VI, during both walking and landing were calculated by use of the following equation [25].

$$ SI=\frac{\left(xop-xno\right)}{\left(1/2\right)\left(xop+xno\right)}\times 100 $$

Where *x*_*op*_ is the mean of a given gait variable for operated limb and *x*_*no*_ is the mean of a given gait variable for non-operated limb. Time between first and second forelimb strike (Tf_1_f_2_) was calculated by subtracting time of contact of the non-operated limb from time of contact of the operated limb. The number of trials where there was a measurable difference in the time of first and time of second forelimb strike (NTf_1_f_2_) was counted. Time between first forelimb and first hindlimb strike (Tf_1_h_1_) was calculated by subtracting time of contact of first forelimb from time of contact of first hindlimb.

#### Rescue analgesia

Observational pain assessments were completed at 3, 6, 12, 24, 30, 36, 48, 54, 60, and 72 h after treatment (Fig. 1) using a subjective scoring system (Additional file 1). Baseline assessments were made for each animal 24 and 48 h before initial and second surgery. A priori, it was decided that any cat that with a pain score of four at any time would be administered rescue analgesia. Pain scores were assigned by a single trained observer. For cats requiring rescue analgesia, meloxicam was administered at a dose of 0.1 mg/kg SC.

### Statistical analysis

Statistical analyses were performed using JMP and SAS (JMP Pro, version 11, SAS 9.4, SAS Institute, Cary NC). Wilcoxon signed-rank test and a paired t-test were used to compare the area under the curve minus baseline (AUC) of pain score versus time between groups. For pain score versus time curves a logistic regression with repeated measures analysis for time and maximum likelihood fitting method was used (PROC GLIMMIX in SAS). Pain score was treated as an ordinal variable in a logistic model with treatment group, period, time as main effects and with interaction terms of time*treatment and period*time (See Additional file 2). The GLIMMIX output also calculated the probability of a score of baseline versus time for both periods and for both treatment groups. A predicted time at which a probability of 0.5 was reached, is similar to an LD50, was obtained (Fig. 3).

Mixed model analysis was conducted to test the effect of time on the outcome measures in the control group in order to see if the cats undergone onychectomy had symmetrical gait. Cat was included as a random effect in the mixed model. Within each treatment a repeated measures analysis was used on the six times (6, 24, 30, 48, 54 and 72 h) with a residual option for the covariance structure (see Additional file 3 for a script of the mixed model parameters and options). Dunnett’s test was used to compare each time point to the baseline value. When walking and landing data were compared between the control group and the ER-Bup group, mixed model analysis was used to test for the effect of treatment on the outcome measures in this crossover design. Cat was included as a random effect in the mixed model (see Additional file 4 for a script of the mixed model parameters and options). Within each treatment a repeated measures analysis was used on the six times (6, 24, 30, 48, 54 and 72 h) with a residual option for the covariance structure. In this analysis, because of a significant period effect, each set of baseline data was separated and the average baseline value of each cat was calculated for each response measure. The difference between this average baseline value was subtracted from the measured response value at each time point in the analysis. Tukey’s test was used to evaluate treatment effects at each time point. A Chi-square for independent test was used for NTf_1_f_2_. The values of *P* < 0.05 were considered significant.

## Results

### Pain score

**Fig. 2:**
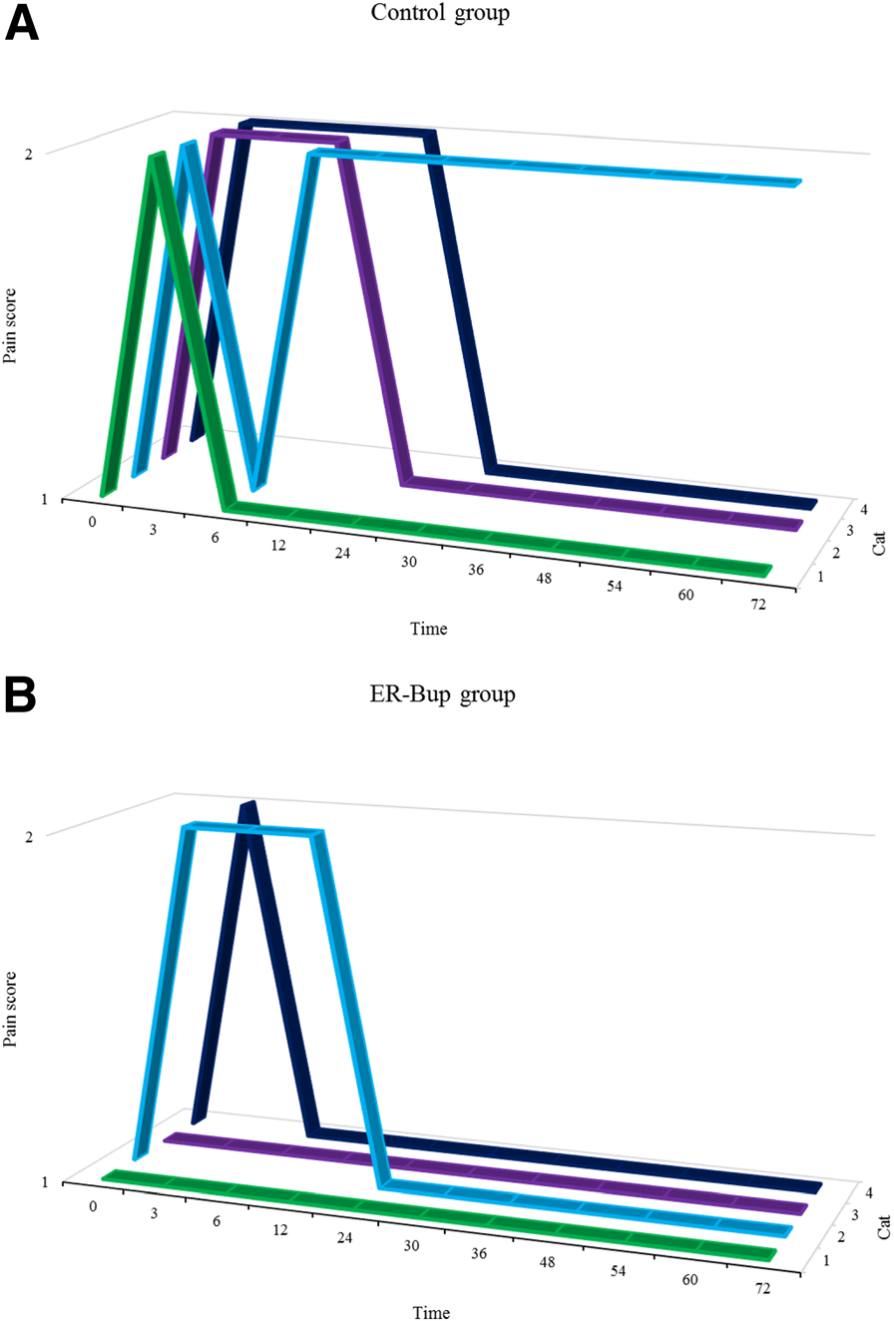
Effect of the treatment on subjective pain score. 3D line graphs demonstrating the subjective pain scores for control (**a**) and extended release formulation of buprenorphine HCL (ER-Bup) groups (**b**) before and after onychectomy. Time point 0 h is the mean of assessments at 24 and 48 h prior to surgery (baseline). Observational pain assessments were performed at 3, 6, 12, 24, 30, 36, 48, 54, 60, and 72 h following surgery

No cats were administered rescue analgesia during study period. No pain scores of greater than two were recorded at any time for any cat (Fig. 2). For each cat, the area under the curve (AUC) of pain score above baseline versus time for both the control and ER-Bup treatment was calculated (Table 2). A paired t-test of AUC for control and ER-Bup group had a *p*-value of 0.0473, while a nonparametric test, Wilcoxon signed rank, gave the minimum possible value for four cats, namely 1/16 (0.063).

**Table 2:** The area under the curve of pain score above baseline versus time

Cat	Control	ER-Bup
1	3	0
2	66	16.5
3	16.5	0
4	28.5	3

*ER-Bup* extended release formulation of buprenorphine HCL

**Fig. 3:**
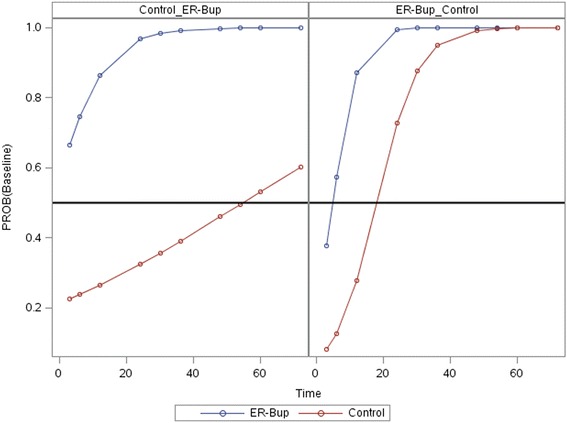
Probability of pain score having a value equal to baseline versus time. The blue line represents the ER-Bup treatment and the red line represents control treatment. The left panel represents the sequence of saline, then ER-Bup injection, while right panel ER-Bup, then saline injection. Horizontal reference line at Probability = 0.5. The predicted time at which a probability of 0.5 is reached, similar to an LD50, is around the first time measurement (3 h) in the ER-Bup group (*blue curves*) in the left and right graphs. For the control group (*red curves*) the time for probability of 0.5 is quite different for the two periods, around 18 for the right graph and 55 for the left graph

The pain scores in Fig. 2 were further examined in a repeated measures logistic model with treatment group, period, and time as main effects and with interaction terms of time*treatment and period*time. Both time and period*time were significant factors (Table 3). The probability of the pain score being at baseline level versus time is shown in Fig. 3. The left panel is for the cats that received the control first and then ER-Bup, while the right panel is for the reverse sequence, ER-Bup then Control. For both panels the blue ER-Bup curves quickly rise toward a high probability of baseline score. The red control curves are both less than the ER-Bup curves and have quite different slopes. The horizontal reference line is a 0.50 probability of baseline score. Both ER-Bup curves reach the reference line before 6 h, while the left control curves has a crossing time around 55 h, while the right control curve crossed around 18 h. The large difference in the crossing times, similar to an LD-50, is the basis for the significant interaction term, period*time.

**Table 3:** ANOVA table for repeated measures analysis of pain score versus time for PROC GLIMMIX

Effect	F value	Pr > F
Period	1.55	0.2686
Treatment	1.53	0.2714
Time	7.77	0.0069
Time*Treatment	1.14	0.2898
Time*Period	4.61	0.0353

The cats were all successfully trained to walk across the PSW, and to jump down onto the PSW. Contemporaneous notes showed that the cats were jumping down onto the PSW after 3 days or training, but required the full 30 days allotted for training to consistently walk across the PSW in a straight line.

### Landing data: comparison to baseline for control group

**Fig. 4:**
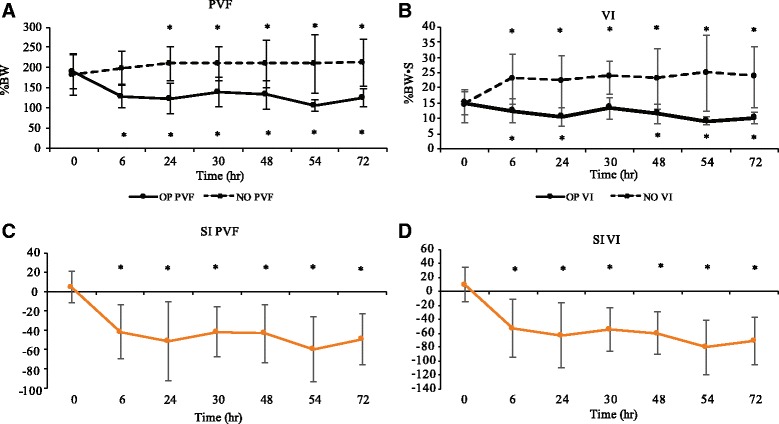
Landing kinetic data for control group. **a** Peak vertical force (PVF) for the operated limb (OP) and non-operated (NO) limb; **b** Vertical impulse (VI) for OP and NO; **c** Symmetry indices (SI) for PVF; **d** SI for VI. Line graphs demonstrating the mean ± SD in control group at various time points before and after onychectomy (*n* = 4). Solid line is OP, dotted line is NO. Time point 0 h is the mean of each gait parameter at 24 and 48 h prior to surgery (baseline). The gait parameters were recorded at 6, 24, 30, 48, 54, and 72 h following surgery. The SI of 0 means there is perfect symmetry between the forelimbs, the value of −200 means the cat is non-weight bearing on the operated limb. * indicates significant difference from baseline in each parameter, shown above upper line and below lower line (<0.05)

All cats satisfactorily completed five trials that could be analyzed through this study, although two baseline trials were missing due to technical errors. The outcome measures calculated for the landing data in control cats are summarized in Table 4 and Fig. 4, and time points that were significantly different from baseline are indicated. PVF and VI were significantly decreased for the operated limb and increased for the non-operated limb compared to baseline, and SI for PVF and for VI were significantly different from baseline in all time points. Tf_1_f_2_ and Tf_1_h_1_ tended to be increased above baseline following surgery (Table 4).

**Table 4:** Kinetic data (mean ± SD) collected from control cats that had undergone a unilateral onychectomy and following landing from an elevated perch

Time	OP PVF (%)	NO PVF (%)	OP VI (%BW/sec)	NO VI (%BW/sec)	SI PVF	SI VI	Tf1f2 (sec)	Tf1h1 (sec)
*P*-value	<0.0001	0.0064	<0.0001	<0.0001	<0.0001	<0.0001	0.0125	<0.0001
0	189.14 ± 41.78	182.59 ± 52.18	15.02 ± 3.74	14.05 ± 5.30	4.86 ± 16.13	9.58 ± 24.58	(−0.0011 ± 0.012)	0.11 ± 0.024
6	129.07 ± 29.58*	197.07 ± 42.10	12.47 ± 3.76*	22.77 ± 8.27*	(−41.42 ± 28.22)*	(−52.93 ± 41.52)*	0.0060 ± 0.0094	0.15 ± 0.053*
24	123.98 ± 37.59*	208.88 ± 41.59*	10.54 ± 3.00*	21.99 ± 8.43*	(−51.01 ± 40.92)*	(−63.33 ± 46.62)*	0.0070 ± 0.0098	0.12 ± 0.014
30	138.75 ± 36.16*	210.07 ± 41.86*	13.27 ± 3.49	23.38 ± 5.44*	(−41.43 ± 25.83)*	(−54.12 ± 31.04)*	0.011 ± 0.010	0.14 ± 0.014*
48	132.88 ± 34.57*	209.55 ± 57.90*	11.51 ± 3.14*	22.79 ± 9.88*	(−43.13 ± 29.78)*	(−59.79 ± 30.42)*	0.012 ± 0.010	0.12 ± 0.020
54	105.87 ± 14.16*	209.88 ± 73.06*	9.04 ± 1.34*	24.72 ± 12.49*	(−59.72 ± 33.38)*	(−80.21 ± 39.60)*	0.018 ± 0.044*	0.12 ± 0.020
72	124.97 ± 23.18*	211.96 ± 58.16*	10.19 ± 1.87*	23.50 ± 9.91*	(−49.35 ± 26.43)*	(−70.77 ± 33.78)*	0.0090 ± 0.010	0.12 ± 0.020

*indicates significant difference from baseline (<0.05)

*OP* Operated limb, *NO* Non-operated limb, *PVF* Peak vertical force, *VI* vertical impulse, *SI* symmetry indices, *Tf1f2* Time between first and second forelimb strike, *Tf1h1* Time between first forelimb and first hindlimb strike

NTf_1_f_2_ was 19 out of 78 trials (24%) in baseline period, and 59 out of 120 trials (49%) at following surgery period in control group (*P* = 0.0045, OR: 2.49).

### Landing data: evaluation of treatment effect

**Fig. 5:**
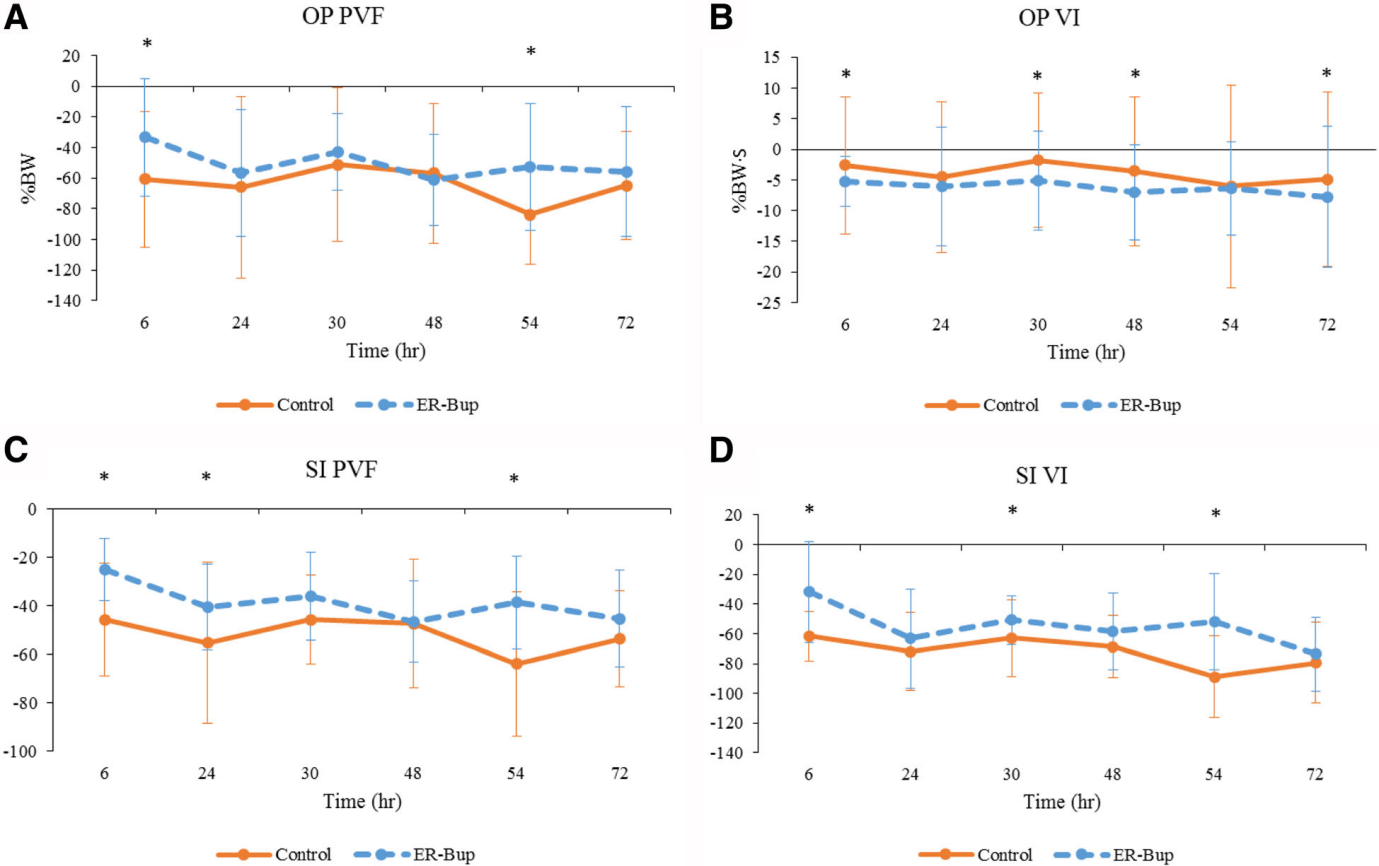
Effect of treatment with ER-Bup on landing kinetic data. **a** Peak vertical force (PVF) for operated limb (OP); **b** Vertical impulse (VI) for OP; **c** Symmetry indices (SI) for PVF; **d** SI for VI. Assessments were made 24 and 48 h initial and second surgery (baseline) and 6, 24, 30, 48, 54, and 72 h after surgery. The average baseline value was subtracted from the measured value at each time point in the analysis. Line graphs showing the changes from baseline in each gait parameter over study period. The mean difference ± SD in control group and extended release formulation of buprenorphine HCL (ER-Bup) group at various time points after onychectomy (*n* = 4). Orange solid line is control group, blue dotted line is ER-Bup group. Negative value of SI means the cat put less weight on operated limb after surgery.* indicates significant difference between groups (<0.05)

The landing data is summarized in Tables 5 and 6 and Fig. 5. There was a significant treatment effect observed for all variables except for the Tf_1_f_2_. ER-Bup resulted in significantly greater PVF on the operated limb and significantly lower PVF on the non-operated limb compared to the control group. ER-Bup resulted in a significantly improved symmetry for PVF. ER-Bup also resulted in a significantly improved symmetry for VI. However, when compared to baseline, there was a greater drop in VI on the operated limb in the ER-Bup group, and a smaller increase in VI on the non-operated limb, compared to control. At first glance, these data are counter to the PVF data, but careful review of the data, including actual values, indicated that following surgery cats in the ER-Bup group had higher PVF values than the control group, but lower contact times (see Discussion). VI is the product of force multiplied by time. Although there was not a significant treatment effect on Tf_1_f_2_, values in the ER-Bup group were smaller, indicating less delay between the two limbs touching the mat compared to the control group. Tf_1_h_1_ (time between first forelimb and first hindlimb touching the mat) was longer in the control group than the ER-Bup group, indicating the hind limbs contacted the ground more quickly following the first contact of the fore limbs in the ER-Bup group compared to the control group.

**Table 5:** Kinetic data (mean ± SD) collected from ER-Bup treated cats that had undergone a unilateral onychectomy and following landing from an elevated

Time	OP PVF (%)	NO PVF (%)	OP VI (%BW/sec)	NO VI (%BW/sec)	SI PVF	SI VI	Tf1f2 (sec)	Tf1h1 (sec)
*P*-value	<0.0001	0.3800	<0.0001	0.0004	<0.0001	<0.0001	0.0002	<0.0001
0	189.57 ± 52.03	173.68 ± 43.23	16.86 ± 5.82	14.75 ± 5.18	7.95 ± 20.60	14.15 ± 31.55	(−0.0005 ± 0.0071)	0.12 ± 0.027
6	156.33 ± 45.68*	180.15 ± 28.05	11.59 ± 3.53*	13.61 ± 2.83	(−17.02 ± 22.82)*	(−17.54 ± 34.80)*	0.0090 ± 0.012*	0.11 ± 0.020
24	132.74 ± 48.23*	177.95 ± 37.77	10.80 ± 3.45*	18.34 ± 6.15*	(−32.71 ± 28.98)*	(−49.02 ± 42.24)*	0.0040 ± 0.0082	0.11 ± 0.017
30	146.57 ± 35.39*	195.21 ± 51.45	11.79 ± 2.45*	17.76 ± 5.46	(−28.07 ± 25.06)*	(−36.69 ± 34.91)*	0.007 ± 0.0098*	0.12 ± 0.025
48	128.38 ± 36.61*	186.89 ± 31.34	9.91 ± 3.01*	15.55 ± 3.61	(−38.66 ± 28.26)*	(−44.44 ± 37.28)*	0.007 ± 0.0098*	0.10 ± 0.026*
54	136.68 ± 46.94*	183.54 ± 52.03	10.45 ± 2.37*	15.95 ± 5.03	(−30.78 ± 29.88)*	(−30.78 ± 29.88)*	0.008 ± 0.010*	0.095 ± 0.017*
72	133.76 ± 47.14*	188.15 ± 28.06	9.12 ± 1.99*	18.49 ± 7.84*	(−37.39 ± 32.35)*	(−59.57 ± 47.28)*	0.011 ± 0.010*	0.097 ± 0.028*

Mean ± SD, *indicates significant difference from baseline (<0.05)

*OP* Operated limb, *NO* Non-operated limb, *PVF* Peak vertical force, *VI* vertical impulse, *SI* symmetry indices, *Tf1f2* Time between first and second forelimb strike, *Tf1h1* Time between first forelimb and first hindlimb strike

**Table 6:** Landing data (summary variables for postoperative time points compared to baseline) for each treatment group and tabluation of treatment effects

		Treatment groups		Treatment effect	
Variable		Control	ER-Bup	*P*-values	Group differences at individual time points
PVF	OP	(−63.97 ± 45.59)	(−50.50 ± 37.44)	0.0026*	ER-Bup > control at 6, 54 h
	NO	23.69 ± 40.23	11.63 ± 37.58	0.0169*	
VI	OP	(−3.89 ± 3.50)	(−6.25 ± 3.22)	<0.0001*	ER-Bup < control at 6, 30, 48, 72 h
	NO	8.99 ± 7.23	1.87 ± 4.50	<0.0001*	ER-Bup < control at all time points
SI	PVF	(−52.03 ± 26.02)	(−38.72 ± 18.63)	<0.0001*	ER-Bup > control at 6, 24, 54 h
	VI	(−72.28 ± 29.47)	(−54.96 ± 27.10)	<0.0001*	ER-Bup > control at 6, 30, 54 h
Tf1f2		0.012 ± 0.021	0.008 ± 0.010	0.076	
Tf1h1		0.014 ± 0.029	(−0.015 ± 0.023)	<0.0001*	ER-Bup < control at all time points

Mean ± SD, *indicates significant difference between groups (<0.05)

*OP* Operated limb, *NO* Non-operated limb, *PVF* Peak vertical force, *VI* vertical impulse, *SI* symmetry indices, *Tf1f2* Time between first and second forelimb strike, *Tf1h1* Time between first forelimb and first hindlimb strike, *ER-Bup* extended release formulation of buprenorphine HCL

NTf_1_f_2_ was 59 out of 120 trials (49%) in the control group, and 48 out of 120 trials (40%) in ER-Bup group. There was no significant difference between the two groups for NTf_1_f_2_ (*P* = 0.15).

### Walking data: comparison to baseline for control group

**Fig. 6:**
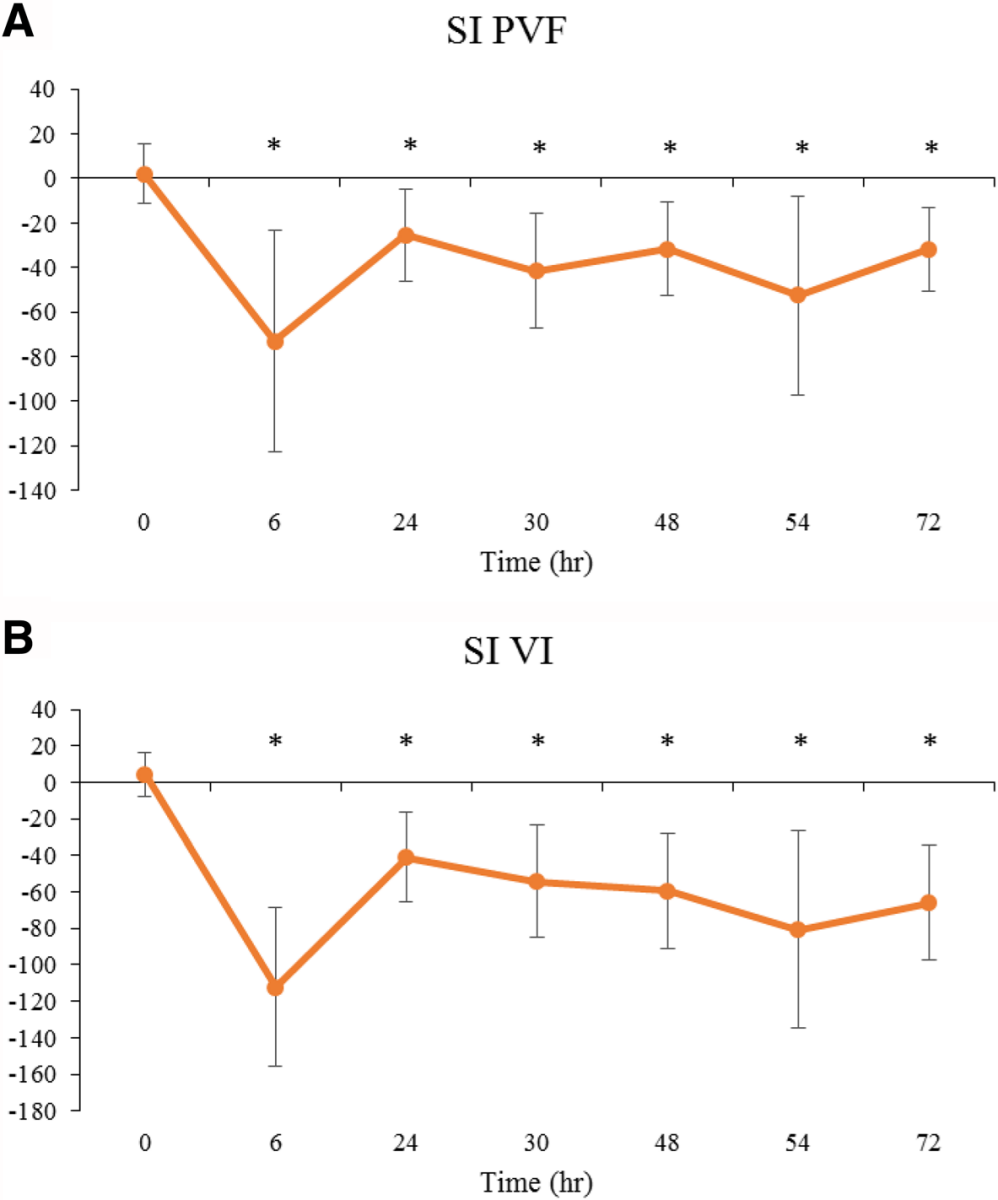
Summary symmetry indices values for kinetic data in the control group during walking. **a** Symmetry indices (SI) for Peak vertical force (PVF); **b** SI for Vertical impulse (VI). Line graphs showing the mean ± SD in control group at various time points before and after onychectomy in four walking cats. Time point 0 h is the mean of assessments at 24 and 48 h prior to surgery (baseline). The gait parameters were recorded at 6, 24, 30, 48, 54, and 72 h following surgery. The SI of 0 means there is perfect symmetry between the forelimbs, the value of −200 means the cat is non-weight bearing on the operated limb. *indicates significant difference from baseline (<0.05)

The cats walked across the PSW at a faster velocity than the a priori target velocity, and so the comfortable speed for traversing the PSW for each cat was considered acceptable. Postoperatively, some cats refused to walk across the PSW. In order to try to minimize the influence of velocity differences on the data, only SI was used for walking data analysis. Overall, cats traversed the walkway at 0.94 ± 0.41 (Mean ± SD) m/s in control group. At least three cats completed four to five trials that could be analyzed at each time point. One cat refused to walk at 6 h following surgery (control group) and another cat (control group) refused to walk except at 6 h after surgery in period 1. The summary values of SI for PVF and VI are shown in Table 7 and Fig. 6, and the time points where values were significantly different from baseline are indicated. SI for PVF and for VI were significantly reduced compared to baseline at all-time points.

**Table 7:** Kinetic data collected during walking in the control group

Time	SI PVF	SI VI
*P*-value	<0.0001	<0.0001
0	2.20 ± 13.43	4.54 ± 12.17
6	(−73.00 ± 49.92)*	(−112.32 ± 43.53)*
24	(−25.39 ± 20.54)*	(−40.87 ± 24.84)*
30	(−41.43 ± 25.83)*	(−54.12 ± 31.04)*
48	(−31.69 ± 21.16)*	(−59.40 ± 31.47)*
54	(−52.48 ± 44.68)*	(−80.71 ± 54.10)*
72	(−31.82 ± 19.04)*	(−65.90 ± 31.35)*

Mean ± SD, *indicates significant difference from baseline (<0.05)

### Walking data: evaluation of treatment effect

**Fig. 7:**
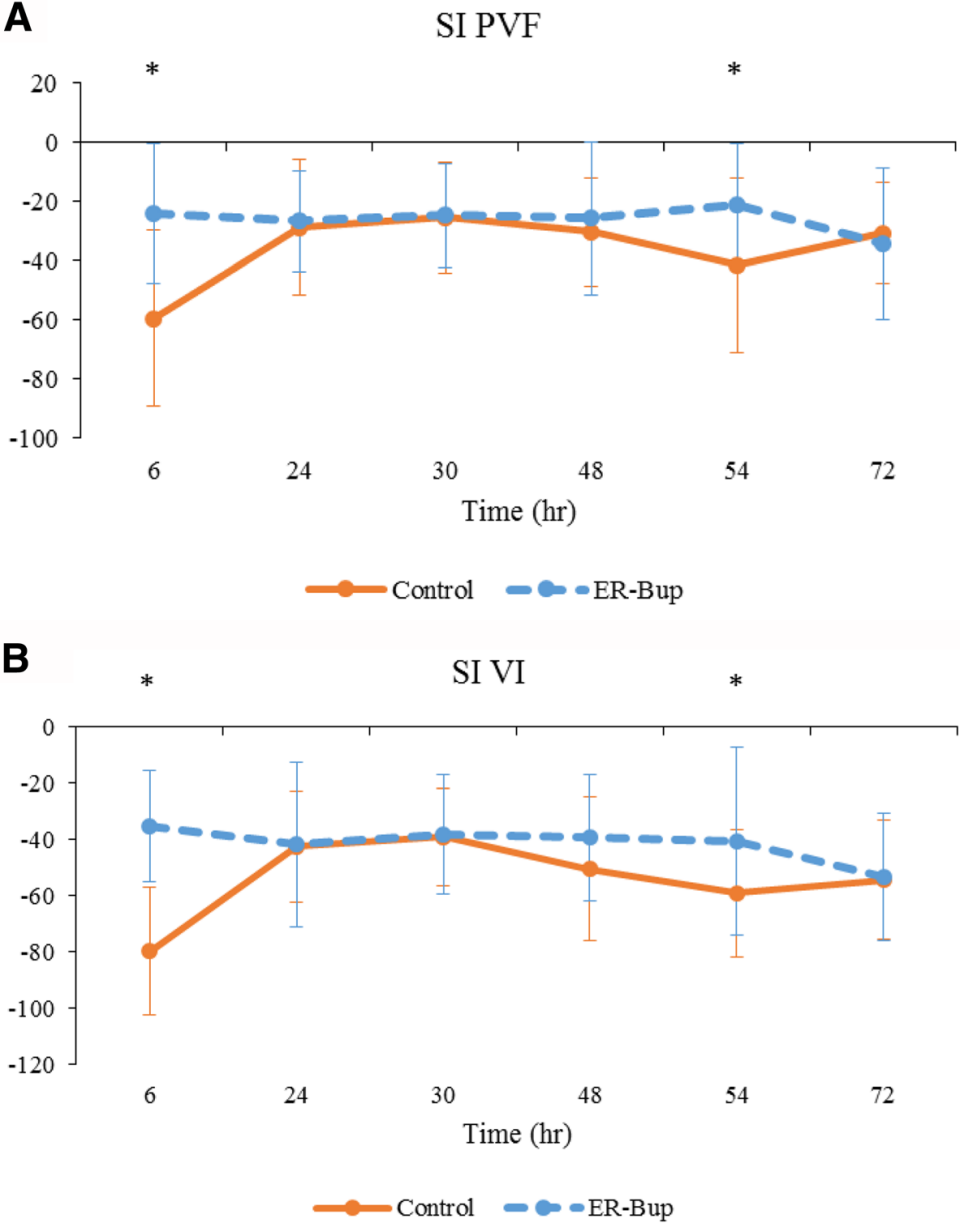
Effect of the treatment on symmetry indices values for kinetic data during walking. **a** Symmetry indices (SI) for Peak vertical force (PVF); **b** SI for Vertical impulse (VI). Assessments were made 24 and 48 h initial and second surgery (baseline) and 6, 24, 30, 48, 54, and 72 h after surgery. The difference between this average baseline value and the measured value were calculated at each time point in the analysis. Line graphs showing the mean difference ± SD in control group and extended release formulation of buprenorphine HCL (ER-Bup) group at various time points after onychectomy (*n* = 4). Orange solid line is control group, blue dotted line is ER-Bup group. Negative value of SI means the cat put less weight on operated limb after surgery.* indicates significant difference between groups (<0.05)

No cats in ER-Bup group refused to walk across the PSW. Cats in ER-Bup group traversed the walkway at 1.16 ± 0.36 m/s, which was significantly faster than the control group (*p* < 0.0001). The summary values of SI for PVF and VI are shown in Table 8 and Fig. 7, and the time points where values were significantly different between groups are indicated. SI for PVF and VI were significantly different between the groups, indicating significantly greater symmetry for these variables in the ER-Bup group.

**Table 8:** Kinetic data (mean ± SD) collected during walking in the control and ER-Bup groups

	Treatment groups		Treatment effect	
Variable	Control	ER-Bup	*P*-values	Group difference in time
SI for PVF	(−54.61 ± 27.98)	(−41.57 ± 22.03)	0.0002*	ER-Bup > control at 6, 54 h
SI for VI	(−36.33 ± 25.59)	(−26.13 ± 21.89)	<.0001*	ER-Bup > control at 6, 54 h

*indicates significant difference between groups (<0.05)

*PVF* Peak vertical force, *VI* vertical impulse, *SI* symmetry indices, *ER-Bup* extended release formulation of buprenorphine HCL

## Discussion

In this pilot study, pre- and postoperative kinetic data were collected from 4 cats by encouraging them to walk freely across a PSW and to jump down on to it (landing data). The present study showed that SC administration of ER-Bup had a positive influence on subjective pain scores, walking and landing kinetic parameters, during 72 h postoperatively. These results should be confirmed in a larger study as the clinical significance of our results is difficult to judge in a small pilot study.

To the authors’ knowledge this is the first report that has used jumping down onto a PSW for evaluation of limb use following surgery. Landing data from cats that had undergone onychectomy could be collected from all cats at all-time points, unlike walking data. In addition, the landing data appeared to provide desired information, and additionally other outcome measures could be collected, such as Tf_1_f_2_, Tf_1_h_1_, and NTf_1_f_2_. These are novel outcome measures that intuitively make sense, especially the time difference between when the first forelimb hits the ground and the second, but they require further investigation as valid measures. The landing data appeared to show the treatment effect in a similar way to the walking data. Additionally, landing data were easier to collect, and could be analyzed more quickly.

The present study highlights the disparity between subjective pain scores and objective evaluations of limb use following onychectomy in cats, and if one accepts that in this situation decreased limb use is an indicator of pain, our data clearly point to the need for development of more sensitive, and valid, subjective measures. Based on our subjective pain scores assigned by trained observers, no cats required rescue analgesics in either group, and the cats looked comfortable at the end of study. No cat scored greater than two out of five at any time during the study, and the direct experience of PDK, DB and BDXL in these studies supports the result that the cats looked comfortable. That is not to say we believe these cats are comfortable, on the contrary, our kinetic data showed large asymmetry in kinetic variables between the operated and non-operated limbs suggesting discomfort associated with the procedure. However, this is very difficult to detect subjectively.

A previous study has suggested an analgesic effect of ER-Bup, however the pain evaluations consisted of two non-validated subjective pain scales [23] and no placebo group. In contrast, our study showed a clear analgesic effect over a 72 h period postoperatively using objective measures and a control group comparison.

However, a weakness of our study is that the subjective scoring system used was not one of the partially validated subjective pain scales currently available [14, 16–18]. This was because the study was performed before most of the information on these scales was published. Additionally, the assessment tools that have been produced thus far have been developed for client-owned cats, not research cats. Although there is no data on this subject, our observations are that research cats are less demonstrative than client-owned cats and other unpublished observations indicate these subjective tools are less sensitive in research cats than client-owned cats, similar to the situation in dogs [22].

Kinetic parameters measured using a PSW in cats during walking have been used to evaluate the effectiveness of analgesics for acute and chronic pain [21, 26, 27]. However, although several reports have evaluated gait symmetry and limb loading in cats, some of them have accepted the data generated even if a limb only contacted the walkway once or twice [26, 28] and wide ranges of velocity are accepted [19]. The present study highlighted the difficulty in collecting walking data from freely moving cats as velocity cannot be easily controlled, and in some cases, cats in the saline treatment group refused to walk. Variation in velocity is a well-known factor that influences the kinetic data during walking [29, 30]. Conversely, landing data are easily collected, although this approach is not likely to be useful to assess hindlimb pain.

Although there were significant differences between baseline and postoperative period in Tf_1_f_2_ and NTf_1_f_2_, significant differences were not observed between the two treatment groups. The coarseness of data collection may have contributed to the lack of difference. The frequency of data acquisition was limited to 60 Hz using the PSW and so a single frame is equivalent to 0.017 s. This frequency of data acquisition might be not fast enough to detect changes in the time between first forelimb and second forelimb strike.

**Fig. 8:**
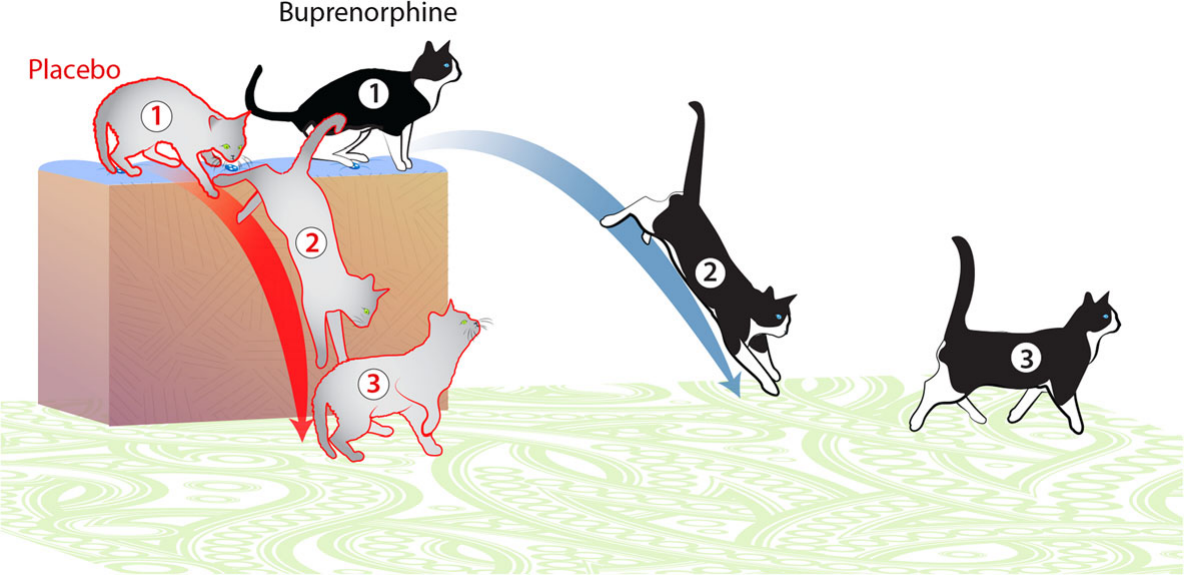
Illustration of the retrospectively observed jumping characteristics of cats in each of the two treatment groups. In the placebo treated cats, they tended to jump down in a vertical line, apparently minimizing the distance of the jump down. They showed anxiousness about jumping (1), appeared to jump down in a very vertical manner (2) and following landing, took longer for the hind limbs to contact the mat, and then they moved away holding the operated paw up (3). The ER-Bup treated cats tended to be less hesitant (1), jumped further away from the platform in a more normal arc (2) and on landing, quickly brought the hind limbs in contact with the mat and moved forward (3)

A significant increase in Tf_1_h_1_ was detected after surgery and values were significantly longer in the control group. At first glance, this is counter intuitive – one would expect that a cat landing from a jump, and with a painful forelimb, would more quickly bring the hindlimbs down to the ground. In order to try to explain this, we retrospectively evaluated the videos captured during the study, and we observed that cats in the control group appeared to jump down more vertically, appearing to be trying to shorten the jump distance, but conversely, appeared to spend longer on their forelimbs following jumping. Cats in the ER-Bup group tended to jump more normally, jumping out, away from the platform, and thus the hindlimbs made contact with the PSW more quickly. These observations are detailed in Fig. 8. However, these, observations need to be evaluated using a higher frequency of data capture and methods to measure the distance from the perch to the point of landing on the PSW.

Surprisingly, landing VI for the operated limb in the control group was significantly closer to baseline than VI in the ER-Bup group even though PVF for operated limb in the control group was significantly lower than in the ER-Bup group. Additionally, landing VI of the ER-Bup group was significantly lower than landing VI of the control group. Lower VI during walking and trotting in dogs has been associated with pain and lameness. Our apparently contradictory results for the landing data appears to be due to the contact time. The contact time for operated limb in the control group was longer than in the ER-Bup group (see Additional files 5 and 6). Vertical impulse is defined as the area under the force by time curve. Thus, if contact time is longer, VI may be larger than in another case where force is higher but contact time is shorter. All our other data indicates a positive benefit from the administration of ER-Bup, and therefore we hypothesize that the reason for the longer contact time for operated limb in the control group may be related to a similar phenomenon in humans – humans prefer lower pain for longer, than higher pain for shorter time periods [31]. As described above, the way the cats chose to jump down from the elevated platform may help explain our data.

Limitations of this study include the very small number of the cats evaluated, and the variations in velocity during walking. The small sample size limits statistical power. To minimize the effect of velocity on PVF and VI, velocity should be restricted to a tight range [29, 30]. We used SI as a means of diminishing the effect of velocity, however, it is not known if symmetry indices are affected by velocity in lame animals and this needs investigation.

## Conclusions

In summary, our hypotheses were supported. Unilateral onychectomy resulted in asymmetry of forelimb kinetic values in cats, and ER-Bup decreased this asymmetry, suggesting SC administration of ER-Bup may be an effective analgesic over a 72-h period postoperatively. Further, data indicate jumping down onto a PSW may be a useful and efficient way of assessing analgesics in cats if a unilateral model of limb pain is used. Further studies are needed to extend our understanding of landing kinetic data in cats.

## Additional files

Additional file 1: Subjective pain scoring system used. (XLSX 10 kb)
Additional file 2: Script of PROC GLIMMIX. (XLSX 22 kb)
Additional file 3: Script of the mixed model parameters and options. (XLSX 15 kb)
Additional file 4: Script of the mixed model parameters and options. (XLSX 15 kb)
Additional file 5: Contact time for operated and non-operated limbs for each group. (XLSX 18 kb)
Additional file 6: Relationship between contact time and Peak vertical force (PVF) in operated limb (OP). (XLSX 18 kb)

## Abbreviations

ER-Bup: Extended release formulation of buprenorphine HCL
NTf_1_f_2_: Number of trials where there was a measurable difference time of first and time of second forelimb strike
PSW: Pressure sensitive walkway
PVF: Peak vertical force
SI: Symmetry indices
Tf_1_f_2_: Time between first and second forelimb strike
Tf_1_h_1_: Time between first forelimb and first hindlimb strike
VI: Vertical impulse

## References

[1] Lascelles D, Waterman A (1997). Analgesia in cats. In Practice.

[2] Robertson SA, Taylor PM (2004). Pain management in cats--past, present and future. Part 2. Treatment of pain--clinical pharmacology. J Feline Med Surg.

[3] Information CH (2013). Fentanyl Patch Can Be Deadly to Children.

[4] Staffieri F, Centonze P, Gigante G, De Pietro L, Crovace A (2013). Comparison of the analgesic effects of robenacoxib, buprenorphine and their combination in cats after ovariohysterectomy. Vet J.

[5] Steagall PV, Monteiro-Steagall BP, Taylor PM (2014). A review of the studies using buprenorphine in cats. J Vet Intern Med/Am Coll Vet Intern Med.

[6] Giordano T, Steagall PV, Ferreira TH, Minto BW, de Sa Lorena SE, Brondani J, Luna SP (2010). Postoperative analgesic effects of intravenous, intramuscular, subcutaneous or oral transmucosal buprenorphine administered to cats undergoing ovariohysterectomy. Vet Anaesth Analg.

[7] Robertson SA, Lascelles BD, Taylor PM, Sear JW (2005). PK-PD modeling of buprenorphine in cats: intravenous and oral transmucosal administration. J Vet Pharmacol Ther.

[8] Wright BD (2002). Clinical pain management techniques for cats. Clin Tech Small Anim Pract.

[9] Hunt JR, Knowles TG, Lascelles BD, Murrell JC (2015). Prescription of perioperative analgesics by UK small animal veterinary surgeons in 2013. Vet Rec.

[10] Williams VM, Lascelles BDX, Robson MC (2005). Current attitudes to, and use of, peri-operative analgesia in dogs and cats by veterinarians in New Zealand. New Zeal Vet J.

[11] Steagall PVM, Taylor PM, Rodrigues LCC, Ferreira TH, Minto BW, Aguiar AJA (2009). Analgesia for cats after ovariohysterectomy with either buprenorphine or carprofen alone or in combination. Vet Rec.

[12] Foley PL, Liang H, Crichlow AR (2011). Evaluation of a sustained-release formulation of buprenorphine for analgesia in rats. J Am Assoc Lab Anim Sci.

[13] Lascelles BD, Dong YH, Marcellin-Little DJ, Thomson A, Wheeler S, Correa M (2012). Relationship of orthopedic examination, goniometric measurements, and radiographic signs of degenerative joint disease in cats. BMC Vet Res.

[14] Brondani JT, Luna SP, Padovani CR (2011). Refinement and initial validation of a multidimensional composite scale for use in assessing acute postoperative pain in cats. Am J Vet Res.

[15] Corbee RJ, Maas H, Doornenbal A, Hazewinkel HA (2014). Forelimb and hindlimb ground reaction forces of walking cats: Assessment and comparison with walking dogs. Vet J.

[16] Calvo G, Holden E, Reid J, Scott EM, Firth A, Bell A, Robertson S, Nolan AM (2014). Development of a behaviour-based measurement tool with defined intervention level for assessing acute pain in cats. J Small Anim Pract.

[17] Holden E, Calvo G, Collins M, Bell A, Reid J, Scott EM, Nolan AM (2014). Evaluation of facial expression in acute pain in cats. J Small Anim Pract.

[18] Brondani JT, Mama KR, Luna SP, Wright BD, Niyom S, Ambrosio J, Vogel PR, Padovani CR (2013). Validation of the English version of the UNESP-Botucatu multidimensional composite pain scale for assessing postoperative pain in cats. BMC Vet Res.

[19] Stadig SM, Bergh AK (2014). Gait and jump analysis in healthy cats using a pressure mat system. J Feline Med Surg.

[20] Lascelles BD, Findley K, Correa M, Marcellin-Little D, Roe S (2007). Kinetic evaluation of normal walking and jumping in cats, using a pressure-sensitive walkway. Vet Rec.

[21] Romans CW, Gordon WJ, Robinson DA, Evans R, Conzemius MG (2005). Effect of postoperative analgesic protocol on limb function following onychectomy in cats. J Am Vet Med Assoc.

[22] Tomas A, Bledsoe D, Wall S, Davidson G, Lascelles BD (2015). Initial evaluation of a canine stifle arthrotomy post-operative pain model. Vet J.

[23] Catbagan DL, Quimby JM, Mama KR, Rychel JK, Mich PM (2011). Comparison of the efficacy and adverse effects of sustained-release buprenorphine hydrochloride following subcutaneous administration and buprenorphine hydrochloride following oral transmucosal administration in cats undergoing ovariohysterectomy. Am J Vet Res.

[24] Swiderski J (2002). Onychectomy and its alternatives in the feline patient. Clin Tech Small Anim Pract.

[25] Abdelhadi J, Wefstaedt P, Galindo-Zamora V, Anders A, Nolte I, Schilling N (2013). Load redistribution in walking and trotting Beagles with induced forelimb lameness. Am J Vet Res.

[26] Romans CW, Conzemius MG, Horstman CL, Gordon WJ, Evans RB (2004). Use of pressure platform gait analysis in cats with and without bilateral onychectomy. Am J Vet Res.

[27] Guillot M, Moreau M, Heit M, Martel-Pelletier J, Pelletier JP, Troncy E (2013). Characterization of osteoarthritis in cats and meloxicam efficacy using objective chronic pain evaluation tools. Vet J.

[28] Verdugo MR, Rahal SC, Agostinho FS, Govoni VM, Mamprim MJ, Monteiro FOB (2013). Kinetic and temporospatial parameters in male and female cats walking over a pressure sensing walkway. BMC Vet Res.

[29] Roush JK, McLaughlin RM (1994). Effects of subject stance time and velocity on ground reaction forces in clinically normal greyhounds at the walk. Am J Vet Res.

[30] Riggs CM, DeCamp CE, Soutas-Little RW, Braden TD, Richter MA (1993). Effects of subject velocity on force plate-measured ground reaction forces in healthy greyhounds at the trot. Am J Vet Res.

[31] Carvalho B, Hilton G, Wen L, Weiniger CF (2014). Prospective longitudinal cohort questionnaire assessment of labouring women’s preference both pre- and post-delivery for either reduced pain intensity for a longer duration or greater pain intensity for a shorter duration. Br J Anaesth.

